# Splice junctions are constrained by protein disorder

**DOI:** 10.1093/nar/gkv407

**Published:** 2015-04-30

**Authors:** Ben Smithers, Matt E. Oates, Julian Gough

**Affiliations:** Department of Computer Science, University of Bristol, Bristol, BS8 1UB, UK

## Abstract

We have discovered that positions of splice junctions in genes are constrained by the tolerance for disorder-promoting amino acids in the translated protein region. It is known that efficient splicing requires nucleotide bias at the splice junction; the preferred usage produces a distribution of amino acids that is disorder-promoting. We observe that efficiency of splicing, as seen in the amino-acid distribution, is not compromised to accommodate globular structure. Thus we infer that it is the positions of splice junctions in the gene that must be under constraint by the local protein environment. Examining exonic splicing enhancers found near the splice junction in the gene, reveals that these (short DNA motifs) are more prevalent in exons that encode disordered protein regions than exons encoding structured regions. Thus we also conclude that local protein features constrain efficient splicing more in structure than in disorder.

## INTRODUCTION

The need for efficient transcription, translation and splicing all place constraints on the amino acid sequence of proteins (1,2). Here we look at the relationship between the intron–exon structure of eukaryotic genes and the protein structure and disorder of the translated product.

Domains are units of protein structure that fold independently. However many proteins, or regions of proteins, do not form a stable three-dimensional structure. These are called intrinsically disordered proteins or intrinsically disordered regions (3). The usage of amino acids is quite different between structured domains and disordered regions (4,5). Certain amino acids may be considered ‘disorder-promoting’, both due to their prevalence within disordered regions and their physical properties. Such amino acids are typically highly flexible, due to the fluctuation of atom positions and backbone dihedral angles (6,7).

Eukaryotic genes are composed of introns and exons. After transcription, introns are spliced out by the spliceosome, leaving the exons to be translated into a protein product. The spliceosome is a complex of snRNAs and proteins, which must first recognise exons and introns (8). Two models for recognition have been proposed, intron-definition and exon-definition, which differ in the unit that the spliceosome first attaches to (9). In both cases recognition requires a variety of sequence features (10). In addition to motifs contained in the intron, such as the branch site and polypyrimidine tract, a number of features are encoded (at least partially) within the exon.

The splice site contains a conserved sequence of nucleotides that, while mostly intronic, extends into exon sequences particularly affecting the first and final two nucleotides of each exon. The canonical splice site has Adenine followed by Guanine prior to the 5′ splice junction, with Guanine after the 3′ splice junction (11). Although this biased composition of the first and final nucleotides of exons has been known for some time (12,13), there appears to have been little investigation into the relationship this has with the amino acid content of the translated protein.

Exonic splicing enhancers (ESEs) are short (∼6 nucleotide) motifs that promote the binding of proteins that regulate the splicing machinery (14). These motifs appear to be diverse—in one analysis, 238 ESEs were identified in the human genome (15). The inclusion of splicing enhancers is thought to be the cause of correlations that exist between amino acid usage and distance from splice junctions: certain amino acids are avoided or preferred with proximity to the splice junction (16–18).

Clear links between protein disorder and splicing have been established. Alternatively spliced exons encode disordered protein regions more than expected, which is suggested to be important for supporting the complexity of multicellular life (19,20). Additionally, bacterial genomes, which lack spliceosomal introns, have reduced levels of disorder compared to eukaryotes (21). Coding regions with low synonymous mutation rates, thought to carry additional functions including splice regulation, have also been shown to be enriched for protein disorder (22). Such observations are particularly interesting as they span the central dogma.

What then, is the relationship between splicing signals and protein sequence and structure? One may expect there to be some conflict between the need to encode signals for efficient splicing and the need to encode a correctly folding protein structure. To address this, we begin by determining the amino acid distribution that is encoded by the biased nucleotide usage in the conserved splice site sequence. We compare this distribution between exons that encode structured protein regions and disordered protein regions. Then, we look again at the correlation between amino acid usage and distance from the splice junction, considering separately structure- and disorder-encoding exons. Finally, we examine how general these results are across the eukaryotic tree using a data set from 91 species, including representatives of the animal, plant, protist and fungal kingdoms.

## MATERIALS AND METHODS

### Data set

Protein sequences, cDNA and the loci of exons for all transcripts of 91 eukaryotic genomes were collected from the Ensembl database (version 63 for genomes taken from the main Ensembl project, version 16 for those from EnsemblGenomes) (23). A list of genomes can be found in Supplementary Table S3.

We discarded exons that do not contribute to the coding sequence, i.e. those entirely part of the 5′ or 3′ UTR. The data set includes over 15 million exons from 1.9 million protein sequence covering animals, plants, fungi and protists.

Exons were mapped to amino acid positions in their corresponding proteins using the genomic coordinates of each exon, relative to the translation start and stop. This approach allows protein sequence annotation to be transferred to the exons that encode the protein.

### Exon classification

Using D^2^P^2^, each exon was classified as disordered if 75% of the amino acids formed by the exon have a 75% consensus prediction of disorder and no amino acids within a SUPERFAMILY-predicted SCOP domain (21,24); exons were classified as structured if 75% of the amino acids are part of a predicted SCOP domain and no amino acids have a consensus disorder prediction.

In addition, each exon was annotated with a start and end phase, indicating where the previous and subsequent introns split codons. The end phase of each exon was calculated by counting (modulo 3) the number of coding nucleotides between the translation start site and the end of each exon; the start phase is given by the end phase of the preceding exon. The first coding exon of each transcript is given a start phase of 0.

### Splice junction amino acid composition

To examine amino acid composition directly at splice junctions, we used the mappings generated between exon locations and amino acid sequences. We computed the composition of amino acids encoded by the last codon in each exon in the data set, taking the codon that spans two exons in the case of a phase 1 or phase 2 intron. For each amino acid, we calculated the fold change between its usage in last residues and its background usage across all protein sequences in all genomes in our data set. When comparing results across different taxonomic groups, the background distribution was recalculated using protein sequences from the genomes in that group only.

To determine if the splice junction distribution was enriched for disordered amino acids, we split the amino acids into two sets: the ten most and least disorder promoting using the TOP-IDP scale (5). We then calculated the percentage of amino acids in each set for both the background and for the last residue of exons. The frequencies were compared using a Chi-square test. To determine if the distribution was skewed more heavily in structure- or disorder-encoding exons, we first identified the set of enriched amino acids across all exons. We then compared the percentage and frequency of amino acids in that enriched set for final residues in structure- and disorder-encoding exons.

### Correlation between amino acid usage and distance from splice junctions

To analyse correlations between amino acid composition and distance from splice junctions, we followed a previously described method to allow for comparison of results. An overview is given here, but we refer the reader to previously published work for full details (16,17).

Briefly, coding sequences are filtered to remove any translations that do not begin and end with start and stop codons, include any premature stop codons, include any uncertain bases or whose length is not a multiple of three nucleotides. Then, the first and last exons for each protein are discarded. Finally, for each exon, any incomplete codons are removed.

To determine how amino acid usage varies with distance from splice junctions, the composition is calculated at a distance from two to 34 residues from the splice site. Spearman's Rank Order Correlation Coefficient is used to correlate the distance and composition. This procedure is performed independently for the start and end of exons, though no residue contributes to composition at both the start and end of an exon.

Five genomes (Cyanidioschyzon merolae, Fusarium oxysporum, Leishmania major, Phytophthora infestans, Ustilago maydis) were not used in this analysis as all exons were removed at the filtering stage. Supplementary Table S3 shows the number of exons in each genome before and after filtering.

### Density of exonic splicing enhancers

In addition, we directly examined the density of ESE motifs in the human genome in exons that encode structured protein regions and exons that encode disordered protein regions. We used the set of ESE hexamers from RESCUE-ESE (15), as well as the INT3 consensus motifs generated by Cáceres and Hurst (25). For each exon, we extracted the DNA that encodes the protein sequence used in the correlation calculations, i.e. codons two to 34 from the splice junction. We then examined all 6-nucleotide sub-sequences and calculated the percentage of these found in each set of ESEs. This procedure was performed using all exons in the filtered set described above and then again for alternative and constituent exons. Constituent exons were identified as those contributing the same coding sequence from the same genetic locus in all transcripts in a gene. Finally, we also compared the lengths of the flanking introns for exons encoding structured regions and exons encoding disordered regions. The mean length of the two flanking introns was calculated for each exon before comparing structure- and disorder-encoding exons using a Welch *t*-test.

## RESULTS

### Splice site nucleotide usage generates a disorder-promoting amino acid distribution

**Figure 1.**
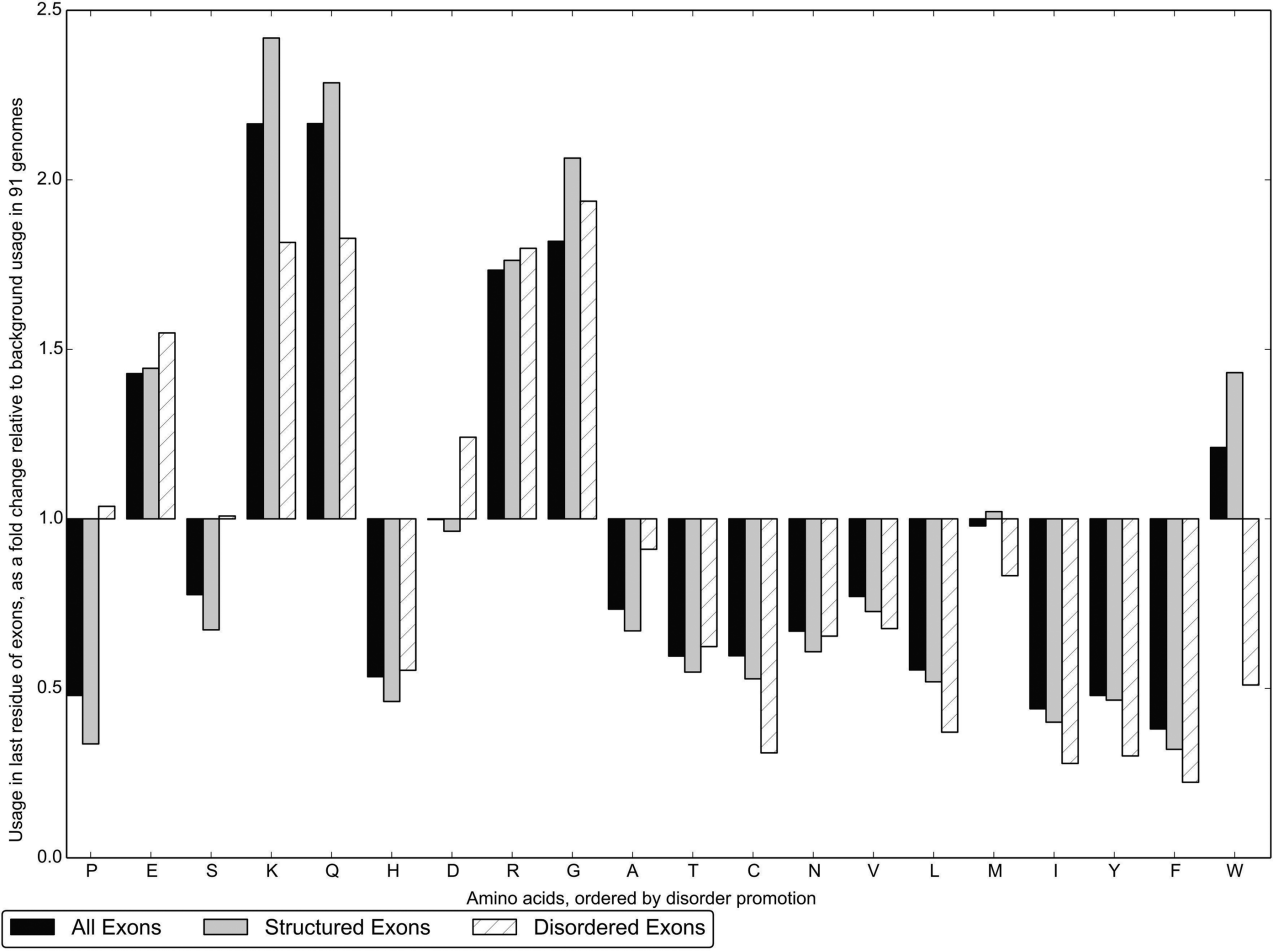
The amino acid usage of the last residue encoded by exons, expressed as a fold change relative to the background distribution of 91 eukaryotic genomes. Results are shown for exons classified as structured (grey; ≥ 75% of amino acids coded by the exon within a SUPERFAMILY-predicted domain, 0 amino acids within a D^2^P^2^ consensus disorder region) or disordered (white, hatched; ≥ 75% of amino acids coded by the exon within a D^2^P^2^ consensus disorder region, 0 amino acids within a SUPERFAMILY-predicted domain). Results for all exons are shown in black. Amino acids are ordered from disorder-promoting to order-promoting (5).

We examined the distribution of amino acid usage for the final residue encoded by each exon and found that there is a large increase in usage of disorder-promoting amino acids. Figure 1 shows the change in composition of the last residue, relative to the background distribution of 91 eukaryotic genomes. The amino acid straddling two exons is used when a codon is split by an intron (i.e. phase 1 or phase 2 introns). In this figure, amino acids are ordered from the most to least disorder-promoting (5). From this ordering, it is apparent that the nucleotide usage at the splice junction produces a distribution of amino acids that increases the use of disorder-promoting residues. The ten most disorder-promoting amino acids account for 74.1% of residues encode by the last codon across all exons, compared to 57.6% in the background distribution (Chi-square test, *P* << 0.0001). This result is robust to the use of other orderings of amino acids that appear in the literature (4,26).

The amino acids enriched across all exons are Glutamic Acid (E), Lysine (K), Glutamine (Q), Arginine (R), Glycine (G) and Tryptophan (W). Lysine and Glutamine show the largest changes, both more than doubling in usage from 5.9% to 12.8% and 4.6% to 9.9% respectively across all exons in the data set.

Exons that encode structured protein regions (grey) and exons that encode disordered protein regions (white, hatched) have a similar distribution of amino acid usage at splice junctions. However, exons encoding disordered regions show less change overall, meaning that the amino acid composition is more biased in structured regions. The six enriched amino acids account for 59.0% of final residues within structure-encoding exons, compared to 52.3% in disorder-encoding exons (Chi-square test, *P* << 0.0001). This finding is supported by nucleotide usage at the splice junction, which we also find to be more biased in exons that encode structure (see Supplementary Table S1). It should be noted that there is increased enrichment for these specific amino acids within structure-encoding exons, rather than an increase in usage of disorder-promoting residues in general. This may be expected, as there is a depletion of the disorder-promoting amino acids that are not encoded by the biased nucleotide composition of the splice site (in particular: Proline, Serine and Aspartic Acid).

**Figure 2.**
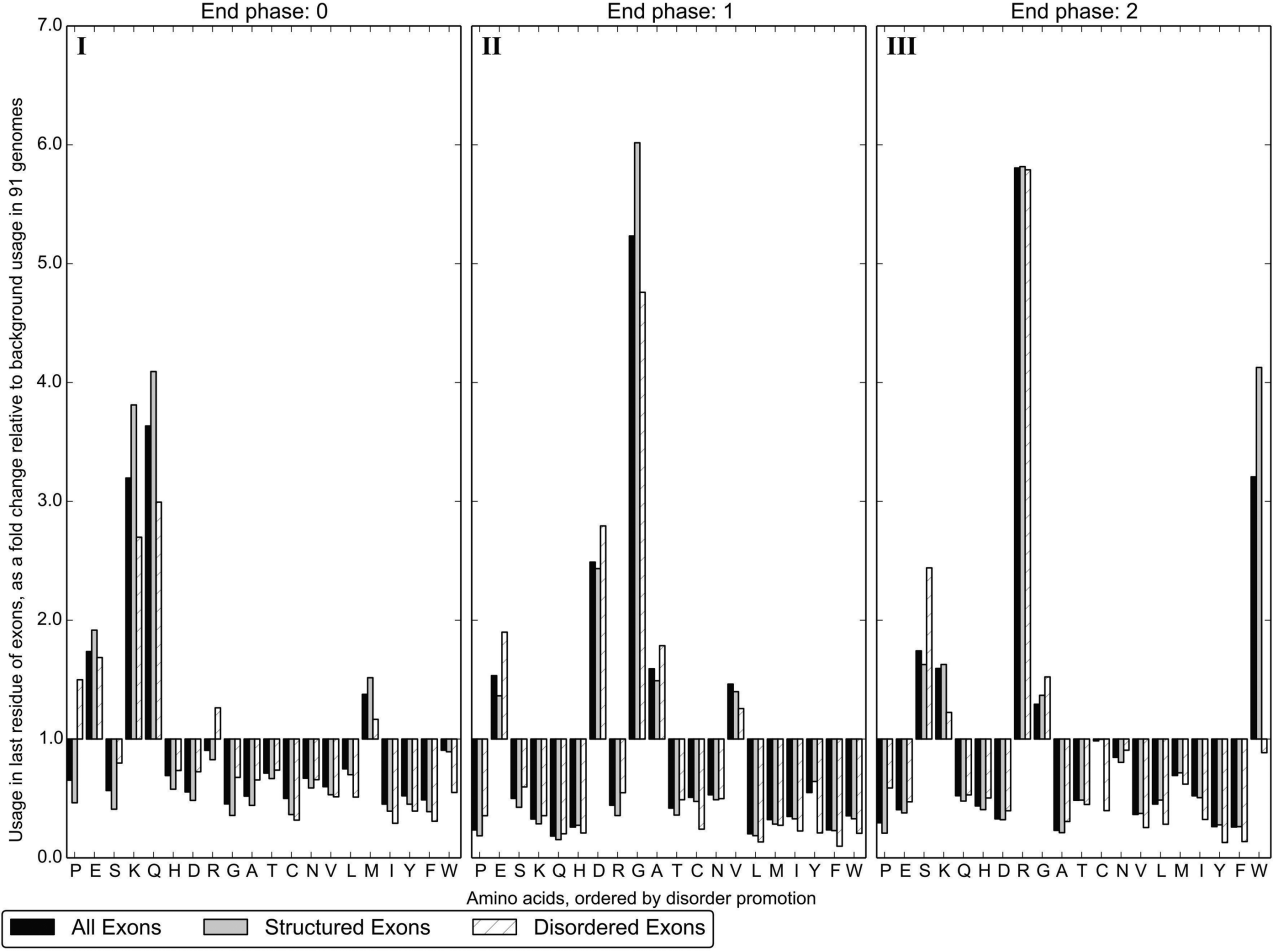
The amino acid usage of the last residue encoded by exons, expressed as a fold change relative to the background distribution of 91 eukaryotic genomes. Results are shown for the three end-phases an exon may have. Colour and classification of exons as structured and disordered is as described in Figure 1.

A distinct distribution is obtained for each of the three end phases an exon may have, which are shown separately in Figure 2. The distribution in Figure 1 may then be thought of as a mixture of three signals.

The amino acid distribution varies for each exon end phase, as this determines the position of the splice junction within a codon and thus dictates how the nucleotide splicing signals translate to amino acids. For example, the most preserved coding nucleotides are *AG* before the 5′ splice site and *G* after the 3′ splice site (11); exons with an end phase of 2 contain all three of these positions as a complete codon. This codon is biased towards *AGG*, which translates to Arginine (R). Thus Arginine is frequently the final amino acid encoded by end phase 2 exons, increasing in usage more than fivefold relative to the overall composition in the genomes (Figure 2.III).

### Amino acid usage at the splice junction is consistent across eukaryotes

**Figure 3.**
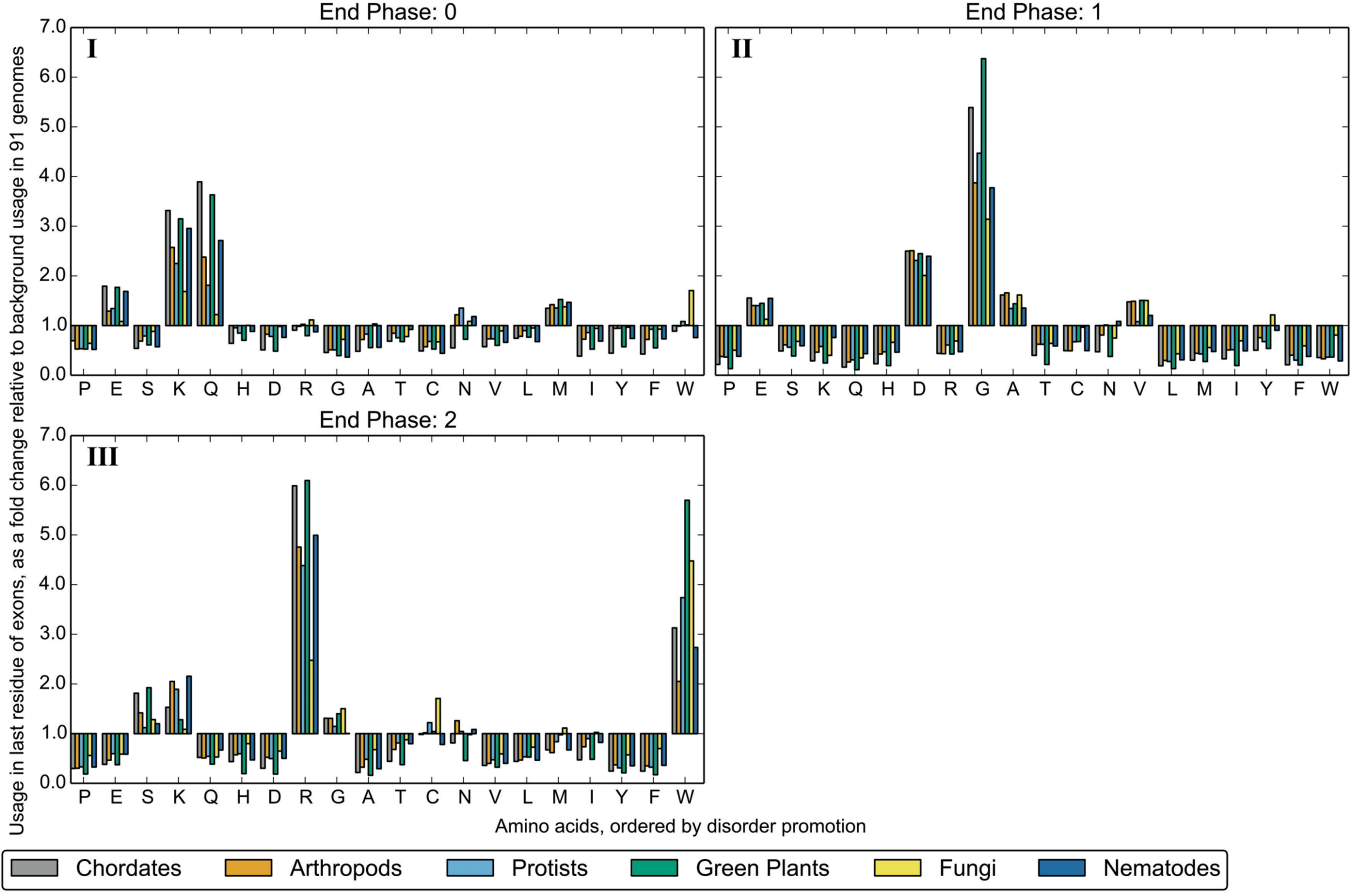
The amino acid usage of the last residue encoded by exons, expressed as a fold change relative to the background distribution. Exons are taken from genomes in six taxonomic groups. The background distribution was calculated separately for each group. Groups are ordered by the number of genomes they contain. Results are shown for the three end phases an exon may have.

We compared amino acid usage at splice junctions in different taxonomic groups and found that the same, mostly disorder-promoting, amino acids show increased usage. Figure 3 shows the change in amino acid usage for the last residue in exons relative to the background usage for six groups: Chordates, Arthropods, Green Plants, Nematodes, Fungi and Protists. The background usage was calculated separately for each group.

Although the distribution is biased towards the same amino acids, the magnitude of changes is larger within Chordates and Green Plants. Fungi in particular show a comparatively modest change in amino acid usage, which may be related to their decreased number of exons (2.4 exons/protein across the 5 Fungal species, compared with 8.2 exons/protein in the other genomes in this study).

### Amino acid usage correlates with distance from splice junctions more strongly in exons encoding disordered protein regions

Previously, Parmley, Warnecke and Wu *et al*. each examined the correlation between amino acid usage and distance from splice junctions, thought to be driven by the inclusion of ESEs (16–18). We extend this work from 30 to 86 genomes (five genomes from our data set were discarded during processing; see materials and methods) and compare exons that encode structured protein regions with those that encode disordered protein regions, as well as considering exon phase. We found that significant preference or avoidance of amino acids near splice junctions is far more common in exons that encode disordered protein regions than those that encode structured protein regions.

**Figure 4.**
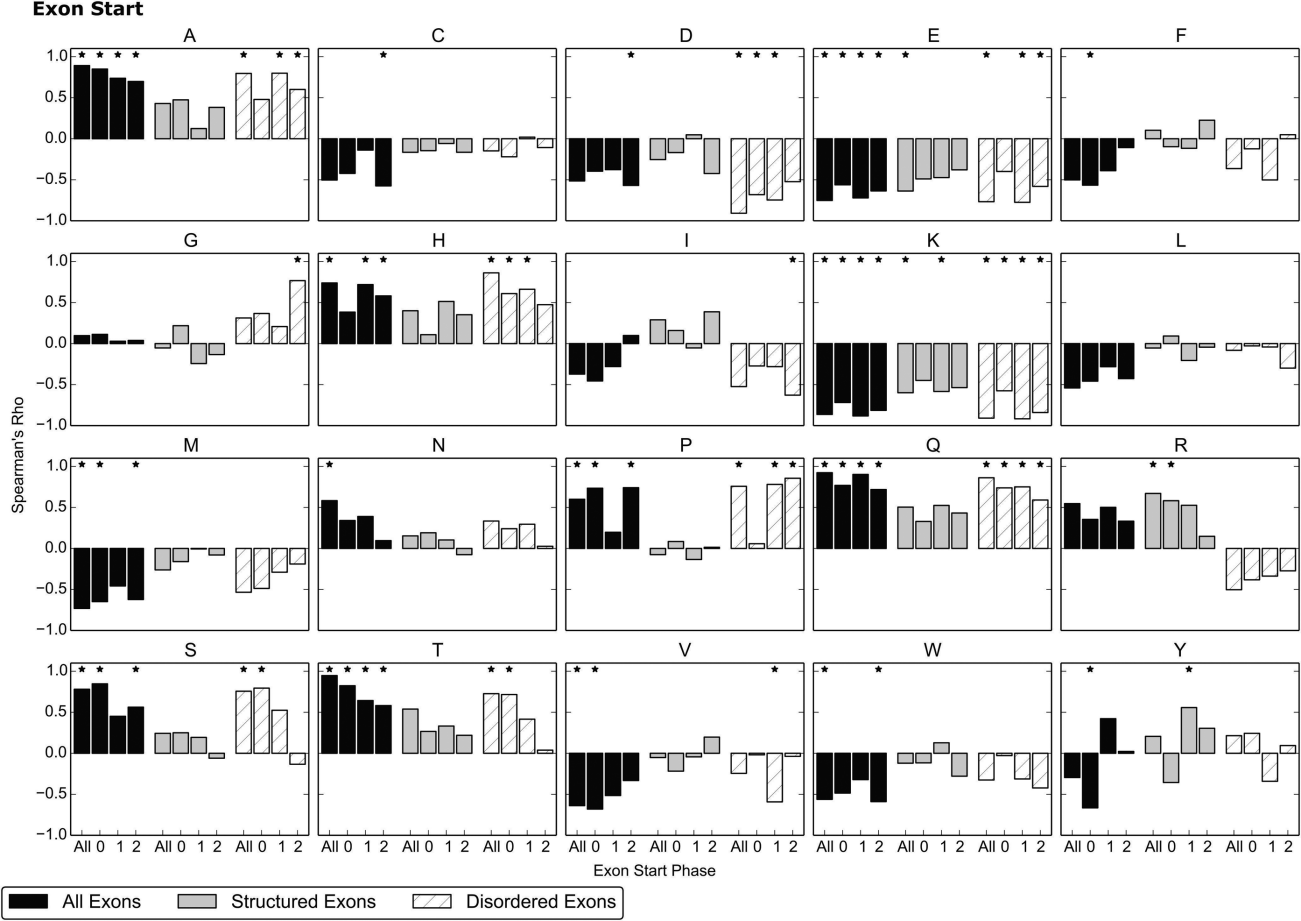
Spearman's Rho values for the correlation in amino acid usage with distance from the splice junction over 33 residues from the start of exons in 86 genomes. Colour and classification of exons as structured and disordered is as described in Figure 1. In addition, results are separated by the start phase of exons in each class. An * denotes a significant value (*P* ≤ 0.001).

**Figure 5.**
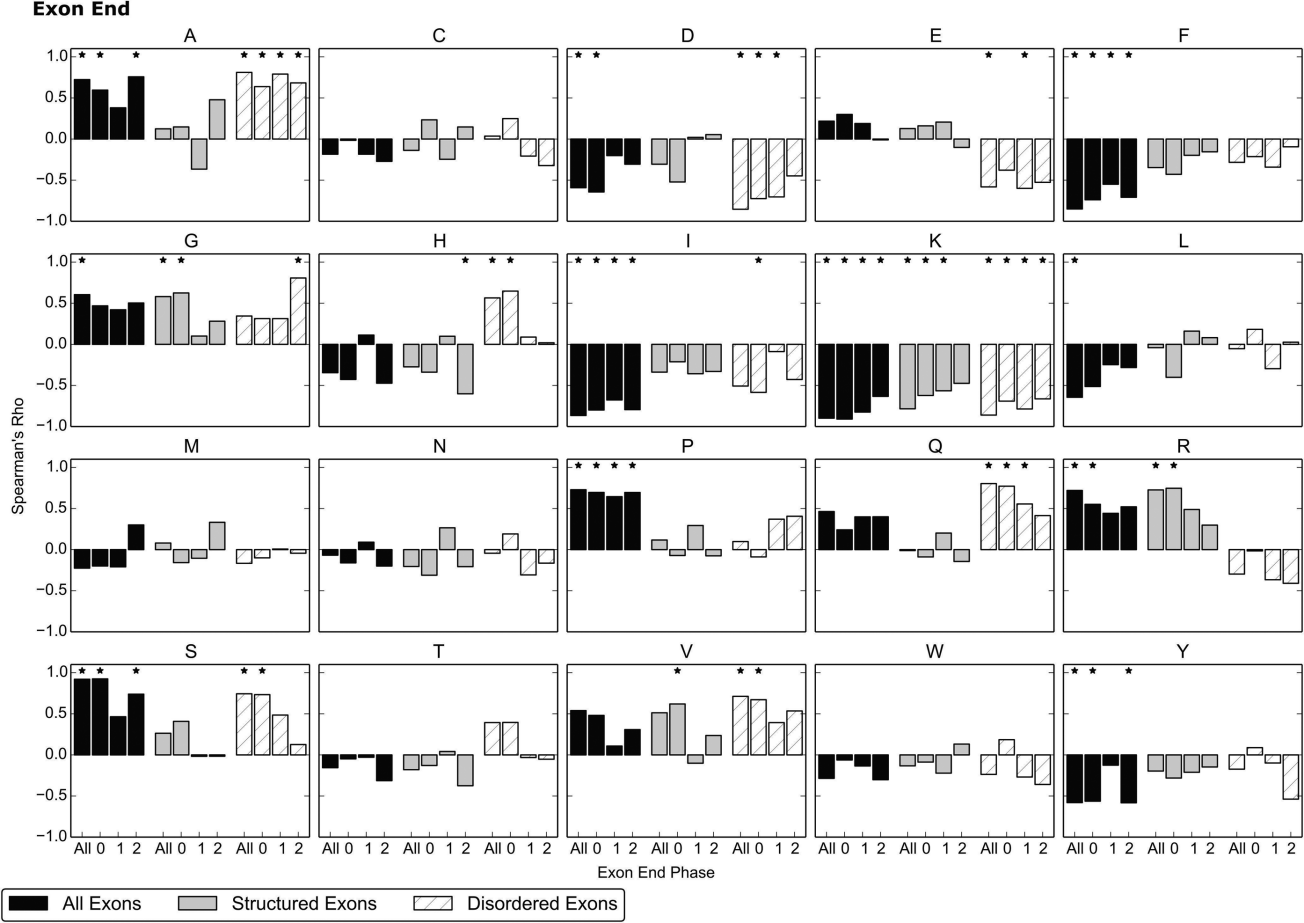
Spearman's Rho values for the correlation in amino acid usage with distance from the splice junction over 33 residues from the end of exons in 86 genomes. Colour and classification of exons as structured and disordered is as described in Figure 1. In addition, results are separated by the end phase of exons in each class. An * denotes a significant value (*P* ≤ 0.001).

Figures 4 and 5 show Spearman's Rank-Order Correlation Coefficients between amino acid usage and distance from splice junctions at the start and end of exons. A positive Rho value indicates increasing usage of an amino acid with distance: the amino acid is avoided near the splice junction.

To aid comparison, we used a similar method to that in previous publications. Supplementary Table S2 compares our results for all exons in the Human genome with previously published results (16); despite differing data sources, qualitatively similar results were obtained.

The amino acids that show a significant change in usage with distance from splice junctions depend on both the phase and the protein region exons encode. For example, the overall avoidance (positive correlation) of Arginine (R) near splice junctions observed both at the start and end of exons is attributable to the trends in exons that encode structured protein regions; disordered exons show the reverse correlation. Additionally, trends observed at the start of exons are not always the same as those at the end: Glutamic acid (E) shows a significant decrease in usage with distance from the start of exons, but a small increase in usage at the end of exons.

The most consistent trend is the increased usage of Lysine (K) near splice junctions, which shows a significant negative correlation in most exon classes at both the start and end of exons. It is interesting to note that Lysine is one of the most disorder-promoting amino acids, somewhat mirroring the preference for disorder-promoting amino acids at the splice junction discussed in the previous section. However, not all amino acids showing increased usage with proximity to splice junctions are considered disorder-promoting.

### Exonic splicing enhancers are found with a higher density in disorder-encoding exons

More amino acids have a significant correlation with distance from splice junctions in exons that encode disordered protein regions than those that encode structured regions. Since these trends are thought to be driven by the inclusion of splicing motifs, we examined the occurrence of known six-nucleotide ESEs in the human genome from RESCUE-ESE (15). We found that these motifs are significantly more common in exons that encode disordered regions than those that encode structured regions. Of all six-nucleotide sub-sequences within 33 codons of the splice junction in exons encoding structure, 10.1% match a known ESE compared to 13.5% in exons encoding disorder (Chi-squared test, *P* << 0.0001).

A number of factors are known to influence the density of ESEs (25), which were examined to rule out the possibility of a confounding interaction with protein disorder. First, we separately compared alternative and constituent exons. In both cases, the significant increase in ESE density within disorder-encoding exons remains (constituent: 9.9% compared to 13.4% in structure and disorder encoding exons respectively; alternative: 10.2% compared to 13.5%). Second, ESE density is positively correlated with intron length. However, we find that exons encoding disordered protein regions are flanked by significantly shorter introns (Welch *t*-test, *P* < 0.0001). Thus exons encoding disordered regions are enriched in ESEs despite being associated with shorter introns. Finally, we consider if the observed differences are related to the use of motifs from RESCUE-ESE. Cáceres and Hurst provide consensus ESE motifs generated from the intersection of four sets of ESEs (25). Here, we compared density using ‘INT3’, a data set requiring motifs to appear in at least three of the four sources. Again, we find significantly more ESE motifs in disorder-encoding exons than structure-encoding (6.7% compared to 4.7%). Since the consensus set contains fewer motifs than RESCUE-ESE (84 in INT3, 238 in RESCUE), it would be expected that the overall density should be lower. However the density relative to the number of motifs is larger within the consensus, which may be indicative of a higher quality set. In addition, we note that the proportional increase in density in disorder-encoding exons is higher when using the consensus set of ESEs.

### Correlation of amino acid usage with distance from splice junctions is *mostly* consistent across eukaryotes

**Figure 6.**
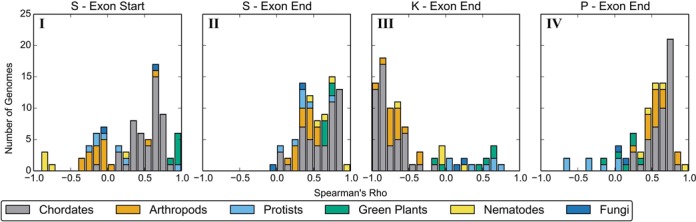
Selected examples of the distribution of rho values for the correlation in amino acid usage with distance from splice junction in exons from six taxonomic groups of eukaryotic genomes. Groups are ordered by the number of genomes they contain. (I) Correlation of Serine usage at the start of exons. (II) Correlation of Serine usage at the end of exons. (III) Correlation of Lysine usage at the end of exons. (IV) Correlation of Proline usage at the end of exons. For full results, see http://bioinformatics.bris.ac.uk/people/ben_smithers/splicing.

We compared the amino acids that are preferred or avoided near splice junctions between six different taxonomic groups and found that most trends are consistent, with some notable exceptions. Figure 6 shows some illustrative examples of the distribution of correlation coefficients for each taxonomic grouping; results for all amino acids and each exon class can be found online at http://bioinformatics.bris.ac.uk/people/ben_smithers/splicing.

With some exceptions, each group displays a similar distribution that is comparable to results for all species discussed above. This suggests a fairly general phenomenon, rather than trends that are local to specific clades of the eukaryotic tree. However, in agreement with previous results (17), nematodes display differing trends to other species—but only at the start of exons (e.g. Figure 6.I compared with Figure 6.II). In addition, the Protists also differ in a number of exon classes, though it should be noted that the group is a disparate paraphyletic assemblage.

In most cases, the distribution of Rho values within the Chordates is approximately normally or half-normally distributed. For most exon classes, the Arthropods and Green Plants appear consistent with this distribution, though with fewer data points in these groups it is hard to draw strong conclusions. However, individual amino acids with different results between these groups are apparent. For example, Serine (S) is typically avoided near the start of exons (positive Rho) in the Chordates; however, Arthropods typically display a negative Rho, indicating increased usage close to the splice junction (Figure 6.I). Similarly, Chordates have a negative correlation with Lysine (K) usage at the end of exons, whereas the Green Plants display either a neutral or positive correlation (Figure 6.III).

## DISCUSSION

We have shown that known nucleotide signals at the splice junction translate to a disorder-promoting amino acid distribution. In addition, the amino acids most enriched by the inclusion of ESEs are Lysine, Glutamic acid and Arginine (16); these are also disorder-promoting. It is important to recognise that forces at the nucleotide-level drive these signals. The biased distribution at the splice site is caused by the need for the spliceosome to recognise splice junctions. Parmey *et al*. provide good evidence that it is the nucleotides of ESE motifs that cause correlations between amino acid usage and distance from splice junctions, rather than an interaction between exons and protein (sub-) structure (16). However, in this work we show both splicing signals correspond to disorder-promoting amino acids. From this, we conclude that the locations of introns within genes are constrained by the tolerance for such residues in the local environment of the protein product. These constraints are significant, yet any amino acid may be found at a given splice junction; overall there is a pressure to accept hydrophilic- and disorder-promoting amino acids that will limit the location of splice junctions, e.g. in the core of a protein domain.

We observe that the canonical splice site is more frequently found in exons that encode structured protein regions than exons encoding disordered regions. Thus the amino acids encoded by the final residue in structured exons display a stronger bias (Figure 1). Conversely, ESE motifs occur more frequently in disorder-encoding exons, meaning more amino acids display a significant correlation between their usage and distance from splice junctions than within exons encoding structure. This suggests that these two classes of exons promote efficient splicing in different ways. Since the presence and conservation of the different splice recognition features is variable (27), it may be that evolution of numerous ESE motifs reduces the selective pressure to maintain the canonical splice site and vice versa. We suggest that it is more favourable for exons encoding protein domains to maintain the canonical splice site rather than evolve an increased number of enhancer motifs as the former impacts fewer amino acids, thus reducing the potentially competing pressures of efficient splicing and the correct folding of the domain. In contrast, exons encoding protein disorder may be more free to include ESE motifs, which may allow otherwise unfavourable mutations of the canonical splice site. In addition, the tolerable inclusion of a larger number of ESE motifs may contribute to higher levels of alternative splicing found in exons encoding disordered regions (3). Alternative splicing is thought to be important for some of the key functions of disordered protein sequence, such as tissue-specific cell signalling and protein interaction networks (28,29). Future work exploring the relationship between splice-signals, disordered protein sequence and alternative splicing may be beneficial for determining causality; weakly conserved splice-signals have previously been associated with alternative splicing (27).

Our analysis included 91 genomes, from a diverse selection of eukaryotic species. The impact of the splice-site on the final amino acid was consistent across these different taxa, though the strength of the signal was variable (Figure 3). Fungi in particular display a comparatively modest bias in amino acid distribution, which is consistent with previous work showing that there is low sequence conservation in the first and last nucleotides of Fungal exons (30). The correlations between amino acid usage and distance from splice junctions that are driven by the inclusion of ESEs are generally consistent across organisms and taxa, though there are some notable exceptions (Figure 6). To explain these variations, we look to differences in splicing behaviour. For example, it has previously been suggested that the differing trends found at the start of exons in Nematodes reflect the prevalence of splice leaders (SL) in these genomes; Warnecke *et al*. hypothesise that different splicing signals are needed to prevent confusion between *cis*-splicing and SL *trans*-splicing (17).

One of the strongest results is the significantly increased usage of Lysine close to the splice junction. However this does not occur in the green plants—Lysine usage instead *decreases* with proximity to the splice junction (Figure 6.III). One possible explanation for this is the relatively high occurrence of alternative splicing by intron retention in plants (31,32). The presence of the polypyrimidine tract causes an over-abundance of Cytosine (C) and Thymine (T) in the last 50 nucleotides of an intron. Thus when an intron is retained, we would expect to see an impact on amino acid composition; in the case of Lysine we would expect it to be under-represented since its codons are *AAA* and *AAG*.

Finally, why does the spliceosome recognise these particular nucleotide signals, which correspond to disorder-promoting amino acids in the translated protein? Although there could be a chance similarity between the nucleotide content of splice signals and disordered residues, we propose an alternative explanation. We hypothesise that the evolutionary changes made possible by the intron–exon architecture of eukaryotic genes, in particular the shuffling, skipping and read-through of exons, will more often take place with break-points in the loops of protein structure. Thus these evolutionary changes will favour the use of blocks of secondary structure, increasing the likelihood of a random change being viable. Perhaps then the splicing machinery has evolved to recognise signals that promote protein disorder because the resulting sampling of protein space through mutation is more likely to survive selection. Evolution itself has evolved to be more efficient.

## SUPPLEMENTARY DATA

Supplementary Data are available at NAR Online.

SUPPLEMENTARY DATA
