# Supplementary material for: Splice junctions are constrained by protein disorder
Source: Nucleic Acids Res. 2015 Apr 30;43(10):4814–22. doi: 10.1093/nar/gkv407 (PMC4446445; doi:10.1093/nar/gkv407)
Supplement: SUPPLEMENTARY DATA [file supp_43_10_4814__index.html]

Splice junctions are constrained by protein disorder — SUPPLEMENTARY DATA 

# Splice junctions are constrained by protein disorder

## SUPPLEMENTARY DATA

**Files in this Data Supplement:**

- SUPPLEMENTARY DATA
