# Supplementary material for: Splice junctions are constrained by protein disorder
Source: Nucleic Acids Res. 2015 Apr 30;43(10):4814–22. doi: 10.1093/nar/gkv407 (PMC4446445; doi:10.1093/nar/gkv407)
Supplement: SUPPLEMENTARY DATA [file supp_gkv407_nar-00219-a-2015-File008.pdf]

Supplementary Material

	Structured				Disordered			
	A	C	G	T	A	C	G	T
-5	28.11%	22.42%	24.41%	25.05%	29.55%	27.58%	27.04%	15.84%
-4	29.43%	22.35%	19.52%	28.70%	32.97%	27.67%	20.47%	18.89%
-3	29.99%	25.47%	21.30%	23.24%	30.20%	27.42%	24.19%	18.19%
-2	32.75%	31.10%	18.10%	18.05%	29.78%	31.27%	18.87%	20.08%
-1	60.04%	11.63%	13.29%	15.04%	57.55%	14.63%	17.59%	10.24%
Last Nucleotide	15.36%	4.90%	70.62%	9.11%	21.75%	7.18%	62.92%	8.15%
Intron								
First Nucleotide	28.04%	13.59%	46.14%	12.23%	35.46%	16.50%	38.50%	9.54%
1	22.56%	17.84%	18.23%	41.37%	24.12%	19.55%	22.25%	34.09%
2	24.95%	22.05%	26.23%	26.77%	23.61%	21.82%	34.77%	19.81%
3	24.89%	23.52%	24.54%	27.06%	26.17%	27.66%	28.17%	18.00%
4	26.15%	23.66%	21.23%	28.96%	30.67%	30.72%	20.12%	18.50%
5	24.13%	24.34%	25.50%	26.03%	26.86%	25.39%	27.88%	19.88%

Table S1: The percentage usage of nucleotides for the first and last six nucleotides of exons across all genomes in this study

	Exon Start						Exon End					
	Parmley *et. al.*		Longest Transcript		All Transcripts		Parmley *et. al.*		Longest Transcript		All Transcripts	
	Rho	P	Rho	P	Rho	P	Rho	P	Rho	P	Rho	P
A	8.66E-01	**1.36E-07**	8.63E-01	**1.05E-10**	7.89E-01	**4.86E-08**	6.61E-01	**4.32E-05**	7.24E-01	**1.89E-06**	6.57E-01	**3.23E-05**
C	9.50E-02	5.90E-01	-1.58E-01	3.81E-01	3.24E-02	8.58E-01	1.40E-01	4.36E-01	-6.68E-03	9.71E-01	-2.07E-02	9.09E-01
D	-4.99E-01	3.50E-03	-4.54E-01	7.98E-03	-4.70E-01	5.83E-03	-5.78E-01	**5.00E-04**	-5.00E-01	3.05E-03	-4.66E-01	6.24E-03
E	-6.42E-01	**8.31E-05**	-6.52E-01	**3.88E-05**	-7.15E-01	**3.00E-06**	1.25E-01	4.80E-01	2.33E-01	1.93E-01	2.65E-01	1.36E-01
F	-5.20E-01	2.00E-03	-5.16E-01	2.11E-03	-4.47E-01	9.19E-03	-7.57E-01	**1.40E-06**	-7.14E-01	**3.14E-06**	-5.72E-01	**5.12E-04**
G	-5.80E-02	7.50E-01	1.63E-01	3.65E-01	9.56E-02	5.97E-01	3.01E-01	8.86E-02	4.21E-01	1.47E-02	3.74E-01	3.19E-02
H	6.07E-01	**2.00E-04**	5.34E-01	1.37E-03	5.16E-01	2.13E-03	-2.02E-01	2.60E-01	-9.22E-02	6.10E-01	7.42E-02	6.82E-01
I	-8.30E-01	**3.54E-07**	-7.88E-01	**5.31E-08**	-6.74E-01	**1.68E-05**	-8.39E-01	**2.88E-07**	-8.77E-01	**2.18E-11**	-8.53E-01	**2.92E-10**
K	-8.81E-01	**6.95E-08**	-9.33E-01	**2.00E-15**	-8.96E-01	**1.93E-12**	-9.36E-01	**0.00E+00**	-9.23E-01	**1.90E-14**	-9.11E-01	**1.92E-13**
L*	2.79E-01	1.15E-01	-3.34E-01	5.76E-02	-3.67E-01	3.55E-02	5.05E-01	3.00E-03	-1.64E-01	3.62E-01	-2.58E-01	1.47E-01
M	-6.28E-01	**1.30E-04**	-8.33E-01	1.78E-09	-6.84E-01	**1.12E-05**	-4.46E-01	9.80E-03	-5.02E-01	2.89E-03	-5.69E-01	**5.47E-04**
N	-5.82E-01	**5.00E-04**	-6.24E-01	**1.05E-04**	-6.06E-01	**1.88E-04**	-5.90E-01	**4.00E-04**	-7.33E-01	**1.23E-06**	-6.54E-01	**3.61E-05**
P	6.17E-01	**1.80E-04**	6.40E-01	**6.05E-05**	6.26E-01	**9.76E-05**	6.60E-01	**4.42E-05**	7.61E-01	**2.73E-07**	7.33E-01	**1.21E-06**
Q	8.74E-01	**9.77E-08**	6.95E-01	**7.33E-06**	6.89E-01	**9.33E-06**	4.40E-01	1.10E-02	1.80E-01	3.17E-01	1.64E-01	3.61E-01
R*	8.75E-01	**9.34E-08**	7.05E-01	**4.73E-06**	5.39E-01	1.21E-03	9.59E-01	**0.00E+00**	7.74E-01	**1.26E-07**	7.58E-01	**3.18E-07**
S*	4.76E-01	5.00E-03	7.03E-01	**5.10E-06**	7.05E-01	**4.73E-06**	4.50E-01	9.10E-03	7.51E-01	**4.78E-07**	7.68E-01	1.78E-07
T	7.23E-01	**4.45E-06**	8.06E-01	**1.46E-08**	8.39E-01	**1.08E-09**	-2.57E-01	1.50E-01	-2.38E-01	1.83E-01	-1.82E-01	3.09E-01
V	-1.75E-01	3.30E-01	-4.05E-01	1.94E-02	-2.65E-01	1.37E-01	3.91E-01	2.50E-02	3.50E-01	4.57E-02	3.07E-01	8.21E-02
W	-6.90E-02	7.10E-01	-1.58E-01	3.79E-01	-1.66E-01	3.55E-01	-1.25E-01	4.90E-01	-1.63E-01	3.64E-01	-1.23E-01	4.95E-01
Y	-5.50E-02	7.59E-01	-2.83E-01	1.10E-01	-1.26E-01	4.86E-01	-3.76E-01	3.30E-02	-3.87E-01	2.59E-02	-3.04E-01	8.49E-02

Table S2: A comparison of rho and p-values using Spearman's Rank-Order Correlation Coefficient between amino acid usage and distance from splice junctions. Results for all exons in the human genome and only exons in the longest transcript of each genome are compared with previously published results (14). Results for amino acids marked with an asterisk (*), are not directly comparable; Parmley *et. al.* split the results for amino acids with 6 distinct codons. Significant results ($p < 0.001$) are marked in bold

Genome	Group	Proteins	Exons	Filtered
Aedes aegypti	Arthropods	17411	67384	28616
Anolis carolinensis	Chordates	18939	182245	34663
Anopheles gambiae	Arthropods	14667	62363	27727
Arabidopsis thaliana	Green Plants	35386	197160	133321
Atta cephalotes	Arthropods	18093	83459	44801
Bos taurus	Chordates	26977	242863	98801
Brachypodium distachyon	Green Plants	31029	167295	111169
Caenorhabditis brenneri	Nematodes	30746	158839	97160
Caenorhabditis briggsae	Nematodes	21961	121722	75838
Caenorhabditis elegans	Nematodes	29872	190305	131246
Caenorhabditis remanei	Nematodes	31531	157779	95839
Callithrix jacchus	Chordates	43792	442188	234184
Canis familiaris	Chordates	25559	247565	80803
Cavia porcellus	Chordates	19774	195241	55955
Choloepus hoffmanni	Chordates	12435	153957	4931
Ciona intestinalis	Chordates	19858	163800	9008
Ciona savignyi	Chordates	20143	200001	13789
Cyanidioschyzon merolae	Protists	4997	5024	0
Danio rerio	Chordates	41478	375002	207102
Dasypus novemcinctus	Chordates	14846	189258	5382
Dipodomys ordii	Chordates	15853	203636	16644
Drosophila ananassae	Arthropods	15070	56309	28991
Drosophila erecta	Arthropods	15048	55966	29029
Drosophila grimshawi	Arthropods	14986	56275	29057
Drosophila melanogaster	Arthropods	21899	106224	65319
Drosophila mojavensis	Arthropods	14595	54668	28307
Drosophila persimilis	Arthropods	16878	58510	27655
Drosophila pseudoobscura	Arthropods	16871	64248	33732
Drosophila sechellia	Arthropods	16471	58022	28344
Drosophila simulans	Arthropods	15415	53317	24873
Drosophila virilis	Arthropods	14491	54761	28483
Drosophila willistoni	Arthropods	15513	56279	28615
Drosophila yakuba	Arthropods	16082	58650	29413
Echinops telfairi	Chordates	16562	225879	10527
Entamoeba histolytica	Protists	8283	10838	538
Equus caballus	Chordates	22641	238378	103415
Erinaceus europaeus	Chordates	14592	192538	9183
Felis catus	Chordates	15048	183976	8251
Fusarium oxysporum	Fungi	17696	47100	0
Gallus gallus	Chordates	22194	215615	72600
Gasterosteus aculeatus	Chordates	27576	281252	80108
Gorilla gorilla	Chordates	27473	276405	140069
Homo sapiens	Chordates	90720	749321	498130
Ixodes scapularis	Arthropods	20486	88775	35594
Leishmania major	Protists	8312	8316	0
Loxodonta africana	Chordates	25622	264002	68202
Macaca mulatta	Chordates	36384	336801	180065
Macropus eugenii	Chordates	15342	209495	8061
Meleagris gallopavo	Chordates	16483	180280	32761
Microcebus murinus	Chordates	16319	204759	15974
Monodelphis domestica	Chordates	32541	327781	74491
Mus musculus	Chordates	54944	478840	331835
Myotis lucifugus	Chordates	20719	194679	66789
Ochotona princeps	Chordates	15993	213180	19778
Ornithorhynchus anatinus	Chordates	26836	249819	37432
Oryctolagus cuniculus	Chordates	23799	242598	62934
Oryza glaberrima	Green Plants	33164	142095	71380
Oryzias latipes	Chordates	24661	246502	59251
Otolemur garnettii	Chordates	15448	215053	9492
Pan troglodytes	Chordates	34142	340197	207387
Phytophthora infestans	Protists	17787	49146	0
Plasmodium berghei	Protists	4881	12791	5269

Plasmodium chabaudi	Protists	5128	13716	5863
Plasmodium falciparum	Protists	5494	14317	5757
Plasmodium knowlesi	Protists	5102	13296	5264
Plasmodium vivax	Protists	5050	12599	4913
Pongo abelii	Chordates	23533	233684	136149
Populus trichocarpa	Green Plants	43968	215847	128923
Pristionchus pacificus	Nematodes	29644	264970	201726
Procavia capensis	Chordates	16101	213360	14092
Pteropus vampyrus	Chordates	17053	216284	39742
Rattus norvegicus	Chordates	32971	323211	182803
Saccharomyces cerevisiae	Fungi	6696	7054	9
Schistosoma mansoni*	None	13171	81069	53076
Schizosaccharomyces pombe	Fungi	5145	10216	2662
Solanum lycopersicum	Green Plants	34675	156944	95605
Solanum tuberosum	Green Plants	56209	202434	53521
Sorex araneus	Chordates	13192	167208	8191
Spermophilus tridecemlineatus	Chordates	14830	206476	7224
Sus scrofa	Chordates	19083	160510	59697
Taeniopygia guttata	Chordates	18191	164186	26544
Takifugu rubripes	Chordates	47841	632403	115784
Tarsius syrichta	Chordates	13662	168408	10250
Tetraodon nigroviridis	Chordates	23118	267870	63949
Toxoplasma gondii	Protists	7988	42491	28354
Tuber melanosporum	Fungi	7496	29021	15161
Tupaia belangeri	Chordates	15462	204625	8108
Tursiops truncatus	Chordates	16598	211362	38727
Ustilago maydis	Fungi	6522	11420	0
Vicugna pacos	Chordates	11793	147318	11609
Xenopus tropicalis	Chordates	22705	240139	84497

Table S3: The genomes used in this study, along with their phylogenetic grouping. The number of proteins, exons and filtered exons is shown. The latter is described in materials and methods. Schistosoma mansoni(*) was not assigned a taxonomic group as there were no other closely-related genomes available.
